# Antirheumatic therapy is not associated with changes in circulating N-terminal pro-brain natriuretic peptide levels in patients with autoimmune arthritis

**DOI:** 10.1371/journal.pone.0253793

**Published:** 2021-06-25

**Authors:** Thao H. P. Nguyen, Morten Wang Fagerland, Gia Deyab, Gunnbjørg Hjeltnes, Ivana Hollan, Mark W. Feinberg, Gro Ø Eilertsen, Knut Mikkelsen, Stefan Agewall

**Affiliations:** 1 Lillehammer Hospital for Rheumatic Diseases, Lillehammer, Norway; 2 University of Oslo, Faculty of Medicine, Institute of Clinical Medicine, Oslo, Norway; 3 Oslo Centre for Biostatistics and Epidemiology, Research Support Services, Oslo University Hospital, Oslo, Norway; 4 Department of Laboratory Medicine, Vestre Viken Hospital Trust, Drammen, Norway; 5 Department of Internal Medicine, Innlandet Hospital Trust, Lillehammer, Norway; 6 Beitostølen Health and Sport Centre, Beitostølen, Norway; 7 Norwegian University of Science and Technology, Gjøvik, Norway; 8 Harvard Medical School, Boston, Massachusetts, United States of America; 9 Division of Cardiology, Brigham and Women´s Hospital, Boston, Massachusetts, United States of America; 10 Faculty of Health Sciences, Department of Clinical Medicine, UIT - The Arctic University of Norway, Tromsø, Norway; 11 Department of Rheumatology, University Hospital of North Norway, Tromsø, Norway; 12 Volvat Medical Center, Lillehammer, Norway; 13 Department of Cardiology, Oslo University Hospital, Ullevål, Oslo, Norway; Scuola Superiore Sant’Anna, ITALY

## Abstract

**Background:**

Patients with autoimmune arthritis (AA) are at increased risk for impaired cardiac function and heart failure. This may be partly due to the effect of inflammation in heart function. The impact of antirheumatic drugs on cardiac dysfunction in AA remains controversial. Therefore, we aimed to examine effects of antirheumatic treatment on serum N-terminal pro-brain natriuretic peptide (NT-proBNP) in AA patients and its relationship to inflammatory markers.

**Methods:**

We examined 115 patients with AA (64 rheumatoid arthritis (RA), 31 psoriatic arthritis and 20 ankylosis spondylitis) starting with methotrexate (MTX) monotherapy or tumor necrosis factor inhibitors (TNFi) with or without MTX co-medication. NT-proBNP (measured in serum by ECLIA from Roche Diagnostics), and other clinical and laboratory parameters were evaluated at baseline, after 6 weeks and 6 months of treatment.

**Results:**

NT-proBNP levels did not change significantly after 6 weeks and 6 months of antirheumatic therapy (p_baseline-6weeks_ = 0.939; p_baseline-6months_ = 0.485), although there was a modest improvement from 6 weeks to 6 months in the MTX only treatment group (median difference = -18.2 [95% CI = -32.3 to -4.06], p = 0.013). There was no difference in the effects of MTX monotherapy and TNFi regimen on NT-proBNP levels. The changes in NT-proBNP after antirheumatic treatment positively correlated with changes in C-reactive protein (CRP) and erythrocyte sedimentation rate (ESR). Baseline NT-proBNP levels were related to baseline CRP and ESR levels, and some other established markers of disease activities in crude analyses.

**Conclusion:**

Circulating levels of NT-proBNP were related to established inflammatory markers at baseline, and the changes in NT-proBNP after antirheumatic treatment were positively related to these markers. Nevertheless, antirheumatic therapy did not seem to affect NT-proBNP levels compared to baseline, even though inflammatory markers significantly improved.

## Introduction

Autoimmune arthritis (AA) such as rheumatoid arthritis (RA), ankylosing spondylitis (AS) and psoriatic arthritis (PsA) are characterised by excess mortality and morbidity from cardiovascular (CV) events, but this association is not fully explained by traditional risk factors [1,2]. Patients with AA may also have evidence of subclinical myocardial damage and are at increased risk of impaired cardiac function and heart failure (HF) [3–5]. Interestingly, the increased occurrence of impaired cardiac function in AA can only partly be explained by the increased occurrence of coronary artery disease/atherosclerosis, and risk factors such as systemic inflammation may crucially be involved in the pathogenesis [6–9]. Cardiac inflammation (probably both in cardiac microvessels and in the myocardium) could partly contribute to impaired cardiac function in AA [6–9]. Cardiac dysfunction in AA can be explained by a process in which proinflammatory cytokines induce coronary endothelial activation leading to stiffness of the myocardium and fibrosis formation, resulting in impaired relaxation of the myocardium and subsequent diastolic dysfunction [10,11].

Brain natriuretic peptide (BNP) is produced by cardiomyocytes mainly in response to their stretch due to volume or pressure overload. On secretion, it splits into biologically active BNP and the remaining N-terminal proBNP (NT-proBNP), which is biologically inactive and more stable than BNP [12]. NT-proBNP is widely used as significant indicator for the clinical diagnosis of HF and cardiac dysfunction [13,14]. Circulating levels of NT-proBNP, and also diastolic dysfunction have been reported to be increased in AA patients without clinical HF compared to control individuals without AA or other inflammatory diseases [15–18]. This might reflect the presence of subclinical cardiac stress and increased myocardial wall tension, which may contribute to HF progression, due to increased secretion of BNP in cardiomyocytes, induced by proinflammatory cytokines [19,20].

The impact of biologics and other disease-modifying anti-rheumatic drugs (DMARDs) on CV risks has been investigated in the literature, more extensively in RA as compared to other inflammatory arthritis. Several studies have addressed that tumor necrosis factor inhibitors (TNFi) therapy might improve the cardiac function by lowering the overall inflammatory state, thus slowing down the process of coronary atherosclerosis and subsequently decreasing the risk of developing HF in AA [21–23]. However, previous studies assessing the effect of TNFi on the cardiac function have shown conflicting results. Some studies showed no effect of TNFi [24,25], while TNFi might negatively influence cardiac function in other studies [26,27]. Nevertheless, the knowledge about NT-proBNP levels in AA and their responsiveness to antirheumatic treatment is still limited, and the effect of DMARDs on cardiac dysfunction in AA remains controversial.

Therefore, the main aim of this study was to examine if antirheumatic treatment in form of Methotrexate (MTX) and TNFi with or without MTX co-medication (TNFi±MTX) affects NT-proBNP levels in AA, and if NT-proBNP levels were related to inflammatory markers.

## Materials and methods

### Patients

A total of 140 patients with active AA were enrolled in the PSARA (Psoriatic arthritis (n = 40), Ankylosing spondylitis (n = 26), Rheumatoid Arthritis (n = 74)) study at Lillehammer Hospital for Rheumatic Diseases between October 2008 and March 2012.

The inclusion criteria for AA group were: age 18–80 years; fulfilling diagnostic criteria for AS by the modified New York diagnostic criteria [28], Modified Caspar criteria for PsA [29] or the American Rheumatism Association 1987 revised criteria for the classification of RA [30]; able and willing to give written informed consent, and to comply with the requirements of the study; clinical indication for starting treatment with TNFi and/or MTX; use of reliable method of contraception for women with childbearing potential; no clinical or laboratory signs of clinically significant infections; no chronic inflammatory disease other than the selected AAs; not pregnant or breast-feeding; no use of prednisolone >10 mg daily for ≥2 previous weeks, or TNFi for ≥4 weeks before the inclusion; no cancer, uncontrolled diabetes, congestive heart failure (New York Heart Association 3–4) [13], recent stroke, central nervous system demyelinating disease or immunodeficiency.

Patients were examined at baseline and after 6 weeks and 6 months of treatment. Of all included patients (n = 140), 115 completed the 6 months follow-up. Reasons for dropout were side-effects (n = 12), insufficient treatment response (n = 11), hepatitis C (n = 1) and loss to follow-up (n = 1).

### Treatment

All patients had active disease, and there was indication for starting treatment, either with MTX and/or TNFi-treatment (adalimumab, etanercept or infliximab). Medicines were administrated in common recommended doses according to clinical judgment performed by a rheumatologist not involved in this study, in accordance with the Norwegian guidelines that adhere to the main international recommendations [31–33].

Based on the guidelines, all patients starting with TNFi regimen had been previously unsuccessfully treated with MTX. Most of these patients continued to use MTX during TNFi treatment due to the standard protocols, in particular to reduce potential immunological side-effects of TNFi [31]. TNFi was the treatment of choice in most patients with axial spondylarthritis who did not sufficiently respond to nonsteroidal anti-inflammatory drugs (NSAIDs) [32]. Among completers, 51 used MTX monotherapy and 64 TNFi±MTX. Doses of treatment were as follows: etanercept 50 mg subcutaneous (sc) injection once a week, adalimumab 40 mg sc injection every other week, infliximab 3–5 mg/kg intravenous injection at baseline, then following standard dosing regimen. MTX doses were 5–25 mg orally once a week.

### Clinical tests

All patients underwent physical examination by a rheumatologist and completed self-reported questionnaires for evaluation of their disease activity and severity adequate for their condition such as Disease Activity Score for 28 joints (DAS28), Medical health Assessment Questionnaire (MHAQ), Physicians’ Global Assessment Score of disease activity (PGA), Patients’ Global Assessment Score of disease activity (PtGA), Bath Ankylosing Spondylitis Disease Activity Index (BASDAI), Bath Ankylosing Spondylitis Functional Index (BASFI) and Bath Ankylosing Spondylitis Metrology Index (BASMI).

Demographic data, medical history, life-style parameters and medication were recorded.

Endothelial function was assessed by the Reactive Hyperaemia Index (RHI) [32], measured by a fingertip plethysmograph (EndoPAT 2000; Itamar). A RH-PAT score of ≤ 1.67 signified endothelial dysfunction, as recommended by the manufacturer, and in accordance with the cut-off value for patients at risk for coronary artery disease, as determined in the study of Bonetti et al. [34]. Plethysmography was performed after fasting for at least eight hours, including non-allowance for smoking.

### Blood samples

Venous blood samples were drawn after fasting for eight hours. Routine hematologic and biochemical tests including C-reactive protein (CRP), erythrocyte sedimentation rate (ESR), white blood cells (WBC), anti-citrullinated protein antibody (ACPA) and Rheumatoid Factor IgM (RF-IgM) were performed at the local hospital laboratory on the day of collection at all visits. ACPA and RF-IgM were determined by enzyme-linked immunosorbent assays (ELISA), (QUANTA liteTM CCP3 IgG ELISA and QUANTA LiteTM RF IgM ELISA, both INOVA Diagnostics, Inc; San Diego; USA).

Additional blood samples for later analyses (including NT-proBNP analysis) were immediately prepared, divided into small aliquots, and stored at −80°C until analyzed. Before analysis of NT-proBNP used in the current study (in April 2020), samples were deep-frozen for median time of 10.4 years [IQR 9.5,10.9], minimal and maximal time of 8.2 and 11.7 years, respectively.

Serum concentrations of NT-proBNP were determined by electrochemiluminescence on a Roche cobas e 411, using the Elecsys proBNP II assay (Roche Diagnostics), which uses two monoclonal antibodies, and recognizes epitopes located in the N-terminal part (1–76) of proBNP (1–108). This assay provides valid measurement of NT-proBNP in a range of 5–35000 ng/L. The interassay variability ranges from 1.8%–4.2% and the intraassay variability ranges from 1.2%–4.6%. The NT-proBNP analysis was performed at Vestre Viken Hospital Trust, Drammen, Norway, in a standardized manner, in random order, by assessors blinded for the clinical data.

The cut-off value for identification and exclusion of chronic HF in adults is 125 ng/L for the used test kit, as determined by the manufacturer, and in accordance to the recommendations from The European Society of Cardiology Guidelines [13]. At this cut-off value, ESC Guidelines state that NT-proBNP has a very high negative predictive value, comprised between 94% and 98%, and a positive predictive value, comprised between 44% and 57% [13].

Thresholds for high NT-proBNP concentrations (97.5th percentile of normal values, as determined by our laboratory) according to age and sex are described in S1 Table.

### Statistical analyses

Continuous data were expressed as median (interquartile range [IQR]) and categorical data by number (percentage) as appropriate. As most continuous variables were not normally distributed, and particularly because the NT-proBNP measurements contained several extreme values, median regression was applied for comparisons between and within groups. Median regression is analogous to linear regression but provides inference about medians instead of means. The median regression models used the Hall-Sheather bandwidth method as the nonparametric density estimator under the assumption that the residuals are independent and identically distributed. Spearman’s rank-order coefficients, with 95% confidence intervals based on the Fisher Z transformation, were calculated to evaluate correlations between NT-proBNP, AA- and CV related variables at baseline, after 6 weeks and 6 months of antirheumatic treatment. The multiple regression models were adjusted for age and gender (as age and gender are known to influence NT-proBNP levels) and for the baseline characteristics that were statistically significantly related to NT-proBNP in simple regression analysis.

A two-tailed probability value of p < 0.05 was considered statistically significant. For statistical calculations we used Stata/SE 16 (StataCorp LC, College Station, TX).

### Ethics

The study was registered in “Clinical Trials” (NCT00902005), and the Norwegian Biobank register (2054). The study protocol was approved by The Norwegian Regional Committee for Medical Research Ethics (S-07377b), and all the research participants gave written informed consent.

## Results

### Baseline characteristics of all the patients

Baseline clinical and cardiovascular characteristics of patients are shown in Tables 1 and 2. Median age of patients was 56 (23–79), and 55.7% were women. 75% of RA patients were women while 80% of AS patients were men. Median disease duration was 2 years, and patients on TNFi-regimen were 3.9 years older than those starting with MTX monotherapy (coefficient = 3.9 [95% CI 1.90 to 5.90], p < 0.001). Disease activity was high with a median DAS28 of > 5 in RA and BASDAI of > 5 in AS. MTX monotherapy was initiated in about half of the RA and PsA patients, but in none of the AS patients as a result of international recommendations for the management of these diseases. 66.1% used NSAIDs and 19.1% used systemic glucocorticoids in addition to DMARDs (Table 1).

**Table 1:** Baseline clinical characteristics.

	All patients	RA	PsA	AS	MTX	TNFi±MTX
	(n = 115)	(n = 64)	(n = 31)	(n = 20)	(n = 51)	(n = 64)
NT-proBNP	49.6 (24.2–108)	77.1 (29.5–127.6)	34.5 (18.7–54.9)	46.5 (24.3–90.1)	54.2 (29.5–123.9)	46.5 (22.7–97.4)
Age (years)	56 (47–62)	57.5 (51.5–63)	50 (43–61)	49 (43.5–59)	56 (49–63)	55 (46–61)
Women, n (%)	64 (55.7)	47 (73.4)	13 (41.9)	4 (20.0)	31 (60.8)	33 (51.6)
RDD (years)	2 (0.1–10)	1.5 (0.1–8)	2 (0.2–13)	2.5 (1-6-5)	0.1 (0.1–3)	3.8 (1.5–12.5)
**Disease activity**						
CRP (mg/L)	7 (3–14)	8 (3–16)	5 (2–10)	7.5 (3–14.5)	8 (3–19)	6.5 (3–12.5)
ESR (mm/h)	13 (6–26)	18.5 (8–29.5)	7.5 (4–16)	9.5 (7–16.5)	14.5 (7–30)	13 (5–24)
WBC (10^9^/L)	7.1 (5.9–8.6)	7.25 (5.9–8.6)	6.35 (4.8–8.1)	7.9 (6.35–8.6)	6.9 (5.7–8.2)	7.3 (5.9–8.65)
RF IgM, n(%)	47 (42.0)	45 (70.3)	0 (0)	2 (11.1)	22 (44.0)	25 (40.3)
ACPA, n(%)	40 (35.4)	39 (60.9)	0 (0)	1 (5.3)	17 (34.0)	23 (36.5)
BASDAI	5.3 (3.7–6.7)	NA	4.3 (3.0–5.9)	5.6 (4.4–7.3)	5.4 (3.4–6.5)	5.1 (3.7–6.8)
BASFI	3.4 (1.9–5.2)	NA	3.2 (1.3–4.4)	4.1 (2.2–5.9)	3.0 (1.3–4.4)	3.8 (2.3–5.5)
BASMI	3 (2–5)	NA	NA	3 (2–5)	NA	3 (2–5)
DAS28	5.1 (4.2–5.5)	5.1 (4.2–5.5)	NA	NA	4.8 (4.3–5.5)	5.1 (4.1–5.5)
PtGA	5.0 (3.1–6.7)	5.2 (3.8–6.7)	4.3 (2.6–5.9)	5.6 (3.1–7.2)	5.2 (3.2–6.4)	4.9 (3.0–7.1)
PGA	3.4 (2,3–4,7)	3.9 (2.8–4.9)	2.3 (1.6–4.1)	3.0 (2.4–4.5)	3.6 (2.7–4.7)	3.1 (2.0–4.7)
MHAQ	0.5 (0.3–0.8)	0.65 (0.28–0.9)	0.4 (0.3–0.7)	0.43 (0.25–0.73)	0.45 (0.3–0.75)	0.5 (0.28–0.85)
**Treatment, n (%)**						
MTX monotherapy	51 (44.4)	34 (53.1)	17 (54.9)	0	51 (100)	0
TNFi±MTX	64 (55.7)	30 (46.9)	14 (45.1)	100 (100)	0	64 (100)
**Comedication,n (%)**						
Beta-blockers	10 (8.9)	5 (7.8)	1 (3.5)	4 (20.0)	4 (8.0)	6 (9.5)
CCB	9 (8.0)	5 (7.8)	2 (7.1)	2 (10.0)	2 (4.0)	7 (11.3)
ACE inhibitors	4 (8.0)	0	2 (6.7)	2 (10.0)	1 (5.9)	3 (9.1)
NSAIDs	76 (66.1)	47 (73.4)	15 (48.4)	14 (70.0)	36 (70.6)	40 (62.5)
Coxibs	1 (0.9)	0	0	1 (5.0)	0	1 (1.6)
Statins	20 (17.4)	12 (18.8)	1 (3.2)	7 (35.0)	7 (13.8)	13 (20.3)
Acetyl salicylic acid	11 (9.6)	6 (9.4)	2 (6.5)	3 (15)	6 (11.8)	5 (7.8)
Warfarin	1 (0.9)	0	0	1 (5.0)	0	1 (1.6)
Glucocorticoids	22 (19.1)	17 (26.6)	3 (9.7)	2 (10)	8 (15.7)	14 (21.9)

All values are given as median (interquartile range), unless otherwise specified.

Abbreviations: RA: Rheumatoid arthritis, PsA: Psoriatic arthritis, AS: Ankylosing spondylitis, MTX: Methotrexate, TNFi: Tumor necrosis factor inhibitors, NT-proBNP: N-terminal pro-brain natriuretic peptide, RDD: Rheumatic disease duration, CRP: C-reactive protein, ESR: Erythrocyte sedimentation rate, WBC: White blood cells, RF-IgM: Rheumatoid actor immunoglobulin M, ACPA: Anti-citrullinated protein antibody, BASDAI: Bath Ankylosing Spondylitis Disease Activity Index, BASFI: Bath Ankylosing Spondylitis Functional Index, BASMI: Bath Ankylosing Spondylitis Metrology Index, DAS28: Disease Activity Score for 28 joints, PtGA: Patient’s Global Assessment Score of disease activity, PGA: Physician’s Global Assessment Score of disease activity, MHAQ: Modified Health Assessment Questionnaire, NSAIDs: Non-steroidal anti-inflammatory drugs, CCB: Calcium channel blockers, ACE inhibitor: Angiotensin converting enzyme inhibitors, NA: Not applicable.

**Table 2:** Baseline cardiovascular characteristics.

	All patients	RA	PsA	AS	MTX	TNFi±MTX
	(n = 115)	(n = 64)	(n = 31)	(n = 20)	(n = 51)	(n = 64)
**CVD, n (%)**						
Heart failure	0	0	0	0	0	0
Etabished CVD	14 (13.3)	8 (12.5)	1 (3.2)	5 (25.0)	5 (9.8)	9 (14.1)
Hypertention	30 (26.1)	17 (26.6)	7 (22.6)	6 (30.0)	9 (17.7)	21 (32.8)
BMI (kg/m^2^)	26.1 (23.5–29.4)	26.1 (23.6–28.5)	26 (23–29)	27.9 (24.2–31.4)	26 (23.6–27.9)	27.2 (23.3–31.8)
Hyperlipidemia	17 (14.8)	11 (17.2)	3 (9.7)	3 (15.0)	9 (17.7)	8 (12.5)
Current smokers	37 (32.2)	20 (31.3)	7 (22.6)	10 (50.0)	16 (31.4)	21 (32.8)
Diabetes	4 (3.5)	3 (4.7)	0	1 (5.0)	0	4 (6.3)
**Endothelial function**						
RHI	1.84 (1.56–2.20)	1.85 (1.55–2.16)	2.04 (1.63–2.35)	1.68 (1.53–1.96)	1.92 (1.56–2.13)	1.82 (1.59–2.26)
ED, n(%)	40 (34.8)	22 (34.4)	9 (29.0)	9 (45.0)	18 (35.3)	22 (34.4)
**Other**						
TC (mmol/L)	5.2 (4.6–5.8)	5.2 (4.6–5.9)	5.3 (4.9–5.7)	5.0 (3.8–5.5)	5.2 (4.5–5.8)	5.2 (4.6–5.8)
LDL_C (mmol/L)	3.2 (2.6–3.9)	3.2 (2.5–4.0)	3.4 (3.0–4.0)	2.8 (2.3–3.4)	3.2 (2.6–3.9)	3.2 (2.7–3.9)
HDL_C (mmol/L)	1.4 (1.1–1.6)	1.4 (1.2–1.6)	1.3 (1.0–1.6)	1.3 (1.1–1.4)	1.4 (1.1–1.5)	1.3 (1.1–1.6)
TG (mmol/L)	1.2 (0.88–1.6)	1.2 (0.95–1.5)	0.95 (0.81–1.7)	1.3 (0.97–1.7)	1.2 (0.83–1.5)	1.2 (0.95–1.6)
HbA1C (%)	5.6 (5.4–5.9)	5.7 (5.5-6-0)	5.6 (5.3–5.8)	5.6 (5.3–5.9)	5.6 (5.4–5.9)	5.6 (5.3–6.0)
FBG (mmol/L)	5.1 (4.8–5.4)	5.1 (4.9–5.4)	5.1 (4.8–5.4)	5.0 (4.9–5.5)	5.2 (5.0–5.4)	5.0 (4.8–5.4)

All values are given as median (interquartile range), unless otherwise specified.

Established CVD defined as previous presence of any of these conditions: Angina pectoris, stroke, myocardial infarction, carotid stenosis, chronic heart failure, percutaneous transluminal coronary angioplasty, aortic aneurysm.

Endothelial dysfunction defined by RHI ≤ 1.67.

Abbreviations: RA: Rheumatoid arthritis, PsA: Psoriatic arthritis, AS: Ankylosing spondylitis, MTX: Methotrexate, TNFi: Tumor necrosis factor inhibitors, CVD: Cardiovascular diseases, BMI: Body mass index, RHI: Reactive hyperemia index, ED: Endothelial dysfunction, TC: Total cholesterol, LDL_C: Low-density lipoprotein cholesterol, HDL_C: High-density lipoprotein cholesterol, TG: Triglyceride, HbA1C: Glycated hemoglobin, FBG: Fasting blood sugar.

Median BMI was about 26 kg/m^2^, median cholesterol was 5.2 mmol/L, 14.8% had hyperlipidemia, one third of all the patients (32.2%) smoked, 3.5% had diabetes mellitus, none of the patients had known/symptomatic HF and 13.3% reported previous CV diseases (CVD) (defined as history of angina, myocardial infarction, heart surgery, percutaneous transluminal angioplasty, cerebrovascular disease, thromboembolism, aortic aneurysm, peripheral artery disease) (Table 2). The PsA group appeared to be a more healthy group than RA, and it also had lower NT-proBNP (Table 1).

### NT-proBNP and AA- and other CV-related risk factors at baseline

Associations between NT-proBNP and selected clinical and laboratory variables at baseline are shown in Table 3.

**Table 3:** Associations between NT-proBNP and selected clinical and laboratory variables at baseline.

	Crude			Multiple median regression		
	Spearman´s rho	95% CI	P-value	Beta	95% CI	P-value
**CRP**						
All	0.18	-0.06–0.41	0.051	**0.61**	**0.006–1.22**	**0.048**
RA	0.13	-0.12–0.37	0.30	0.49	-0.63–1.61	0.39
PsA	0.22	-0.15–0.54	0.24	**6.19**	**4.96–7.42**	**< 0.0001**
AS	0.03	-0.47–0.42	0.90	-0.15	-2.12–1.83	0.88
MTX	**0.34**	**0.06–0.56**	**0.019**	1.18	-0.43–2.78	0.15
TNFi±MTX	0.06	-0.19–0.30	0.63	-0.15	-0.81–0.52	0.67
**ESR**						
All	**0.24**	**0.06–0.41**	**0.009**	0.43	-0.28–1.14	0.24
RA	0.19	-0.06–0.42	0.13	0.41	-0.58–1.40	0.41
PsA	0.15	-0.22–0.48	0.43	-0.17	-3.67–3.34	0.92
AS	-0.07	-0.50–0.38	0.77	-0.27	-3.79–3.24	0.87
MTX	**0.44**	**0.19–0.64**	**0.001**	0.60	-0.42–1.61	0.25
TNFi±MTX	0.08	-0.17–0.32	0.54	-0.3	-1.43–0.83	0.60
**DAS28 (in RA)**						
RA	0.25	-0.07–0.53	0.12	9.33	-14.4–33.0	0.43
MTX	**0.55**	**0.13–0.80**	**0.015**	17.7	-33.0–68.5	0.47
TNFi±MTX	-0.01	-0.45–0.43	0.96	7.99	-44.3–28.3	0.65
**MHAQ**						
All	**0.22**	**0.04–0.39**	**0.021**	3.21	-26.6–33.0	0.83
RA	0.11	-0.14–0.35	0.39	16.8	-33.9–67.6	0.51
PsA	0.08	-0.28–0.43	0.65	-20	-60.5–20.6	0.32
AS	**0.53**	**0.11–0.79**	**0.016**	-8.72	-18.3–16.5	0.92
MTX	0.09	-0.19–0.36	0.51	20.9	-31.5–73.4	0.43
TNFi±MTX	**0.30**	**0.06–0.57**	**0.015**	1.33	-39.6–42.2	0.95

Bold values denote statistical significance at the p < 0.05 level.

Abbreviations: RA: Rheumatoid arthritis, PsA: Psoriatic arthritis, AS: Ankylosing spondylitis, MTX: Methotrexate, TNFi: Tumor necrosis factor inhibitors, NT-proBNP: N-terminal pro-brain natriuretic peptide CRP: C-reactive protein, ESR: Erythrocyte sedimentation rate, DAS28: Disease Activity Score for 28 joints, MHAQ: Modified Health Assessment Questionnaire.

Median baseline NT-proBNP was 49.6 ng/L [IQR 83.8]. Total, 21 (18.1%) patients presented an NT-proBNP > 125 ng/L which is the cut-off generally set for chronic HF, but only 7 (6.1%) patients had a value above the cut-off value for chronic HF estimated from the distribution of NT-proBNP at the reference laboratory according to age and gender (S1 Table).

In crude analyses, NT-proBNP levels positively correlated with ESR and MHAQ in the total patient cohort, and with CRP and DAS28 in patients treated with MTX monotherapy. However, only association with CRP remained significant after adjustment for age and gender (Table 3).

**Fig 1:**
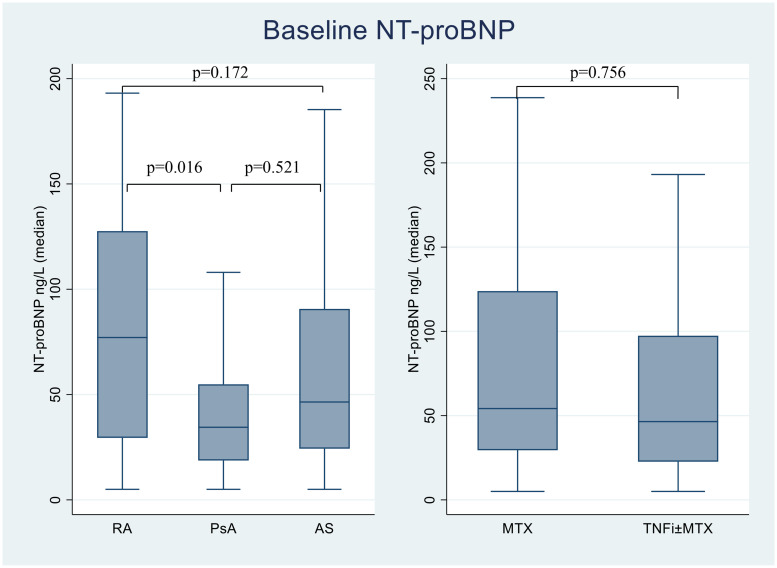
NT-proBNP at baseline. Boxplots displaying the baseline values of NT-proBNP distributed according to diagnosis groups and therapy groups. The lines inside the boxes show the median, bottom and top of the box represent 25 and 75 percentile and whiskers represent minimum and maximum values. Values are given in median. NT-proBNP: N-terminal pro-brain natriuretic peptide, RA: Rheumatoid arthritis, PsA: Psoriatic arthritis, AS: Ankylosing spondylitis, MTX: Methotrexate, TNFi: Tumor necrosis factor inhibitors.

There was no significant difference in NT-proBNP levels between female and male patients, or between patients treated with MTX monotherapy and those treated with TNFi±MTX. No difference in circulating levels of NT-proBNP was observed between ACPA-positive and ACPA-negative patients (data not shown). PsA patients had statistically significantly lower baseline NT-proBNP compared to RA patient (median 34.5 versus 77.1 ng/L; p = 0.016, Fig 1).

### Changes in NT-proBNP levels

**Fig 2:**
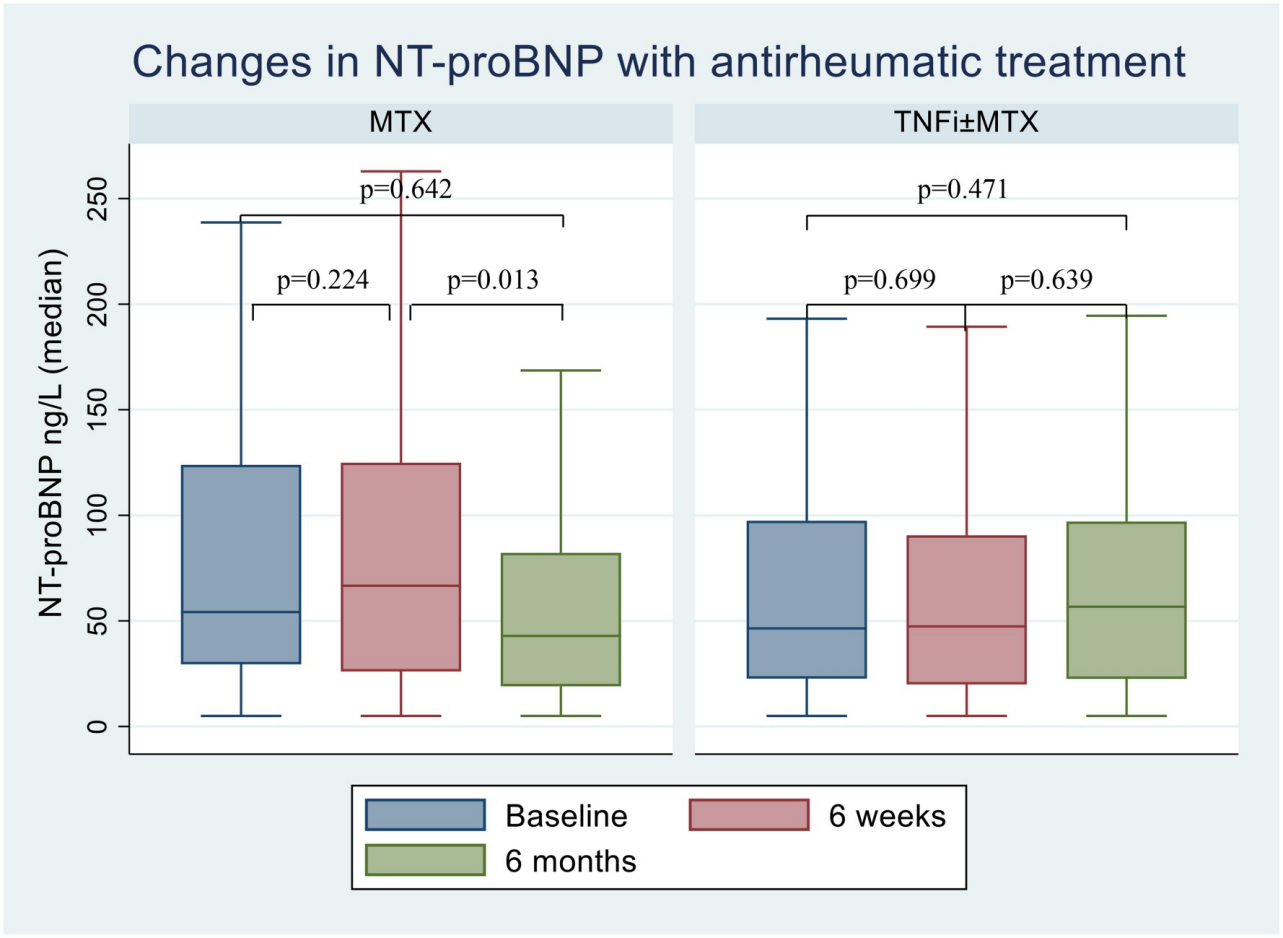
Changes in NT-proBNP with MTX monotherapy and TNFi±MTX treatment. Boxplot displaying the difference in NT-proBNP at baseline and post-treatment with MTX monotherapy and TNFi±MTX for the whole population. The lines inside the boxes show the median, bottom and top of the box represent 25 and 75 percentile and whiskers represent minimum and maximum values. Values are given in median. NT-proBNP: N-terminal pro-brain natriuretic peptide, MTX: Methotrexate, TNFi: Tumor necrosis factor inhibitors.

NT-proBNP did not change significantly after 6 weeks or 6 months of antirheumatic therapy. However, there was a modest significant reduction in NT-proBNP from 6 weeks to 6 months in the MTX only treatment group (median difference = -18.2 [95% CI = -32.3 to -4.06], p = 0.013) (Fig 2). When accessing diagnostic groups separately, we found no significant changes in NT-proBNP levels at any point of time (data not shown).

There was no statistically significant difference in the effect of MTX monotherapy and TNFi±MTX treatment on NT-proBNP levels in RA and PsA patients (comparison not applicable for AS because all AS patients were treated with TNFi alone).

Changes in NT-proBNP and some selected markers of diseases activity are shown in Table 4.

**Table 4:** Changes in selected variables during treatment.

	baseline	6 w	6 m	P(baseline-6w)	P(6w-6m)	P(baseline-6m)
**NT-proBNP (ng/L)**						
All patients	49.6	54.2	45.2	0.94	0.65	0.49
RA	77.1	78.0	66.8	0.27	0.11	0.68
PsA	34.5	41.7	33.7	0.52	0.89	0.70
AS	46.5	27.4	36.8	0.38	0.93	0.66
MTX	54.2	66.6	42.9	0.22	**0.013**	0.64
TNFi±MTX	46.5	47.4	56.7	0.70	0.64	0.47
**CRP (mg/L)**						
All patients	7	2	2	**<0.001**	1.00	**<0.001**
RA	8	2	2	**0.006**	1.00	**0.001**
PsA	5	3	2	0.18	1.00	0.062
AS	7.5	1	1	0.097	1.00	**0.005**
MTX	8	4	2	**0.016**	0.07	**0.027**
TNFi±MTX	6.5	1	2	**0.012**	1.00	**0.003**
**ESR (mm/h)**						
All patients	13	8	7	**<0.001**	1.00	**<0.001**
RA	18.5	10.5	9.5	**<0.001**	1.00	**<0.001**
PsA	7.5	6.5	6	0.097	1.00	0.18
AS	9.5	3.5	3	0.16	1.00	0.16
MTX	14.5	12	9	**0.004**	**0.008**	**0.034**
TNFi±MTX	13	11.5	7	**0.002**	1.00	**<0.001**
**DAS28**						
RA	5.1	3.4	2.7	**<0.001**	**0.024**	**<0.001**
MTX	4.8	3.7	2.7	**0.003**	0.066	**<0.001**
TNFi±MTX	5.1	3.2	2.4	**<0.001**	0.22	**<0.001**
**MHAQ**						
All patients	0.5	0.25	0.2	**<0.001**	**<0.001**	**<0.001**
RA	0.65	0.3	0.2	**0.023**	0.13	**<0.001**
PsA	0.4	0.3	0.25	0.058	1.00	**0.001**
AS	0.43	0.23	0.2	**0.031**	0.30	**0.024**
MTX	0.45	0.28	0.15	**0.007**	0.29	**<0.001**
TNFi±MTX	0.5	0.25	0.2	**<0.001**	0.056	**<0.001**
**PGA**						
All patients	3.4	2	1.4	**<0.001**	**<0.001**	**<0.001**
RA	3.9	2.3	1.4	**<0.001**	**0.003**	**<0.001**
PsA	2.3	1.7	1.3	**0.042**	**0.002**	**0.012**
AS	3	1.8	1.1	**0.002**	0.43	**0.006**
MTX	3.6	2.4	1.8	**<0.001**	**0.017**	**<0.001**
TNFi±MTX	3.1	1.8	1.2	**0.001**	**0.001**	**<0.001**
**PtGA**						
All patients	5	2.6	1.6	**<0.001**	**<0.001**	**<0.001**
RA	5.2	2.9	1.5	**<0.001**	**0.03**	**<0.001**
PsA	4.3	2	2	**0.045**	0.88	**0.033**
AS	5.6	3	1.6	**0.005**	0.067	**0.009**
MTX	5.2	3	2	**<0.001**	0.17	**<0.001**
TNFi±MTX	4.9	2.5	1.5	**<0.001**	**0.001**	**<0.001**

Bold values denote statistical significance at the p < 0.05 level.

Abbreviations: RA: Rheumatoid arthritis, PsA: Psoriatic arthritis, AS: Ankylosing spondylitis, MTX: Methotrexate, TNFi: Tumor necrosis factor inhibitors, NT-proBNP: N-terminal pro-brain natriuretic peptide CRP: C-reactive protein, ESR: Erythrocyte sedimentation rate, DAS28: Disease Activity Score for 28 joints, MHAQ: Modified Health Assessment Questionnaire, PtGA: Patient’s Global Assessment Score of disease activity, PGA: Physician’s Global Assessment Score of disease activity.

### Associations of changes in NT-proBNP with changes in markers of arthritis activity

Associations of changes in NT-proBNP with changes in selected clinical and laboratory variables after 6 weeks and 6 months of antirheumatic treatment are shown in Tables 5 and 6, respectively.

**Table 5:** Associations between changes in NT-proBNP and changes in selected markers of arthritis activity after 6 weeks of treatment.

	Crude			Multiple median regression		
	Spearman´s rho			Beta	95% CI	P-value
**ΔCRP**						
All	**0.27**	**0.09–0.43**	**0.005**	**0.83**	**0.33–1.33**	**0.001**
RA	**0.29**	**0.05–0.50**	**0.02**	1.05	-0.04–2.14	0.059
PsA	0.35	-0.01–0.63	0.059	**3,44**	**2.49–4.38**	**< 0.001**
AS	0.11	-0.35–0.52	0.66	-0.24	-0.97–0.50	0.51
MTX	0.28	-0.001–0.52	0.052	**2.89**	**1.80–3.99**	**< 0.001**
TNFi±MTX	0.22	-0.07–0.47	0.081	0.34	-0.09–0.78	0.12
**ΔESR**						
All	0.08	-010–0.26	0.38	0.43	-0.22–1.09	0.19
RA	0.09	-0.16–0.33	0.46	0.42	-0.66–1,50	0.44
PsA	0.20	-0.17–0.52	0.28	0.13	-1.66 1.92	0.88
AS	-0.03	-0.47–0.42	0.89	-0.50	-2.08–1.08	0.51
MTX	0.08	-0.20–0.36	0.56	0.78	-0.53–2.10	0.24
TNFi±MTX	0.02	-0.22–0.27	0.86	0.04	-0.78–0.85	0.93
**ΔDAS28 (in RA)**						
RA	**0.34**	**0.03–0.60**	**0.032**	17.9	-6.55–42.5	0.15
MTX	0.43	-0.03–0.74	0.086	22.7	-33.6–79.0	0.4
TNFi±MTX	0	-0.39–0.49	0.78	3.55	-40.2–47.3	0.87
**MHAQ**						
All	0.14	-0.05–0.31	0.15	8.31	-14.8–31.4	0.45
RA	**0.25**	**0.01–0.47**	**0.046**	23.9	-12.8–61.0	0.20
PsA	-0.10	-0.44–0.26	0.58	-19.1	-86.0–47.9	0.56
AS	0.08	-0.37–0.51	0.73	10.3	-72.4–93.0	0.80
MTX	0.11	-0.18–0.38	0.45	4.51	-38.3–47.8	0.84
TNFi±MTX	0.16	-0.09–0.39	0.21	12.6	-29.9–55.1	0.56

Bold values denote statistical significance at the p < 0.05 level.

Δ indicates change from baseline to 6 weeks.

Abbreviations: RA: Rheumatoid arthritis, PsA: Psoriatic arthritis, AS: Ankylosing spondylitis, MTX: Methotrexate, TNFi: tumor necrosis factor inhibitors, NT-proBNP: N-terminal pro-brain natriuretic peptide, rho: Spearman’s rho, CRP: C-reactive protein, ESR: Erythrocyte sedimentation rate, DAS28: Disease Activity Score for 28 joints, MHAQ: Modified Health Assessment Questionnaire.

**Table 6:** Associations between changes in NT-proBNP and changes in selected markers of arthritis activity after 6 months of treatment.

	Crude			Multiple median regression		
	Spearman´s rho	95% CI	P-value	Beta	95% CI	P-value
**ΔCRP**						
All	**0.30**	**0.12–0.46**	**0.002**	**0.78**	**0.43–1.14**	**< 0.001**
RA	**0.35**	**0.11–0.55**	**0.005**	**1.26**	**0.69–1.83**	**< 0.001**
PsA	0.29	-0.09–0.59	0.13	**1.56**	**0.76–2.36**	**< 0.001**
AS	-0.03	-0.47–0.42	0.90	0.31	-0.16–0.77	0.19
MTX	**0.32**	**0.04–0.55**	**0.026**	**1.56**	**0.91–2.21**	**< 0.001**
TNFi±MTX	**0.29**	**0.05–0.50**	**0.02**	0.14	-0.32–0.60	0.54
**ΔESR**						
All	**0.26**	**0.08–0.43**	**0.006**	**0.77**	**0.26–1.28**	**0.004**
RA	**0.29**	**0.05–0.51**	**0.019**	**0.74**	**0.16–1.31**	**0.013**
PsA	0.27	-0.11–0.58	0.16	0.59	-1.35–2.54	0.54
AS	0.15	-0.32–0.55	0.53	0.7	-1.33–1.53	0.094
MTX	0.25	-0.04–0.49	0.09	**1.09**	**0.16–2.02**	**0.023**
TNFi±MTX	**0.26**	**0.02–0.48**	**0.037**	0.31	-0.44–1.06	0.41
**ΔDAS28 (in RA)**						
RA	**0.52**	**0.24–0.72**	**0.0007**	14.7	-0.54–29.9	0.058
MTX	**0.73**	**0.42–0.89**	**0.0004**	22	-203–247	0.84
TNFi±MTX	**0.46**	**0.02–0.75**	**0.043**	**17.9**	**1.58–34.3**	**0.034**
**MHAQ**						
All	**0.19**	**0.01–0.36**	**0.039**	16.4	-7.3–40.1	0.17
RA	0.18	-0.07–0.41	0.15	23.8	-14.7–62.4	0.22
PsA	0.17	-0.21–0.50	0.37	25.4	-29.9–80.8	0.35
AS	0.18	-0.29–0.57	0.45	-3.8	-62.9–55.3	0.89
MTX	0.06	-0.22–0.33	0.69	13.3	-41.9–68.5	0.63
TNFi±MTX	**0.29**	**0.04–0.50**	**0.022**	**39.1**	**7.4–70.8**	**0.017**

Bold values denote statistical significance at the p < 0.05 level.

Δ indicates change from baseline to 6 months.

Abbreviations: RA: Rheumatoid arthritis, PsA: Psoriatic arthritis, AS: Ankylosing spondylitis, MTX: Methotrexate, TNFi: Tumor necrosis factor inhibitors, NT-proBNP: N-terminal pro-brain natriuretic peptide, rho: Spearman’s rho, CRP: C-reactive protein, ESR: Erythrocyte sedimentation rate, DAS28: Disease Activity Score for 28 joints, MHAQ: Modified Health Assessment Questionnaire.

There was observed a weak association between changes in NT-proBNP and changes in CRP levels after 6 weeks of antirheumatic treatment (Table 5). Changes in NT-proBNP after 6 months of antirheumatic treatment were statistically significant associated with changes in CRP and ESR in the total patient cohort and with changes in DAS28 in RA patients (Table 6).

## Discussion

Our data showed a statistically significant association between baseline NT-proBNP and systemic inflammatory markers like CRP and ESR, and the changes in NT-proBNP after antirheumatic treatment positively correlated with changes in these markers, which is in accordance with findings of other studies [15–17,35]. The results showed an association between inflammation and cardiac function in term of NT-proBNP in individuals without clinical HF, and might support the hypothesis that inflammation contributes to arterial stiffness and subsequently to an increased ventricular load [6,10].

However, in contrast to inflammatory and clinical parameters, initiation of MTX or MTX combined with an TNFi in RA and PsA patients and TNFi monotherapy in AS patients, was not related to reduction in circulating levels of NT-proBNP during our 6-month follow-up study. One may expect that reduction of inflammation in AA patients would lower circulating NT-proBNP levels, but a direct effect of antirheumatic therapy on NT-proBNP could not be ruled out in the present study. There are few explanations for this instance.

First, this could have been due to the low prevalence of cardiac dysfunction in term of NT-proBNP (only 7 av 115 had increased NT-proBNP value compared to the reference healthy population), and the median values for serum NT-proBNP at baseline is low, indicating a normal cardiac wall tension. Therefore, improvement of NT-proBNP may have been underpowered.

Second, as the development of HF is a progressive, chronic process before symptom onset, it is also possible that the duration of follow-up of 6 months was too short a time period to uncover impactful changes on levels of NT-proBNP. Moreover, we cannot rule out that NT-proBNP changes would become manifest if antirheumatic therapy occurred at later pre-symptomatic stages of left ventricular dysfunction at a time when NT-proBNP levels are known to be higher. The impact of therapy in patients with the highest quartile of NT-proBNP levels compared to the lowest would be of future interest to explore in larger studies.

Third, although the main regulatory mechanism for synthesis and secretion of BNP is distension of ventricular cardiomyocytes, several mechanisms, such as neuroendocrine activation and systemic inflammation, may ultimately contribute to upregulation of BNP in myocardium [6,36]. Fish-Trotter et al. found cytokines activated during the inflammatory process, were associated with high circulating levels of NT-proBNP independent of the underlying causes, suggesting that BNP are differently regulated in disorders involving inflammation [37,38]. Given that the BNP have been shown to have inflammatory and protective immunomodulatory effect [39,40], we may expect that in patients with AA, increased BNP production and secretion is a protective mechanism to maintain inflammatory homeostasis. This effect may explain why antirheumatic therapy did not seem to affect the circulating levels of NT-proBNP as compared to baseline, even though inflammatory markers significantly improved.

Thus, in contrast to some previous studies which showed the cardioprotective effect of anti-inflammatory treatment [21–23], our results do not support the notion that these specific antirheumatic therapies protects against HF in AA. Importantly, our findings go against the likelihood that TNFi treatment has a negative effect on cardiac function in patients with AA in the absence of HF. The results might be important and clinically relevant given the adverse history of TNFi studies in patients with HF [26,27]. Our findings suggest that inhibition of excess TNF in AA patients in the absence of HF does not increase ventricular stress and overload.

Furthermore, in this study we found that circulating levels of NT-proBNP were significantly lower in patients with PsA compared to RA patients. There is ample evidence that RA and PsA exhibit independent risks of HF. Recently, Ahlers et al. revealed that patients with RA were at 21% greater risk of HF independent of traditional CV risk factors, compared to non-RA controls [41]. In another meta-analysis in PsA, there were observed a 31% increase in the risk of HF in patients with PsA compared to the general population [4]. A study by Fernandez-Gutierrez et al. [42] directly compared CVD, defined as angina, myocardial infarction, peripheral vascular disease and/or stroke, in patients with RA and PsA, and found significantly lower odds of CVD in patients with PsA (n = 1147) compared to RA patients (n = 2152) (odds ratio 0.46 [95% CI 0.23 to 0.92], p = 0.028). Nevertheless, in a large observational cohort of 5315 patients with RA, PsA and spondylarthritis [43], the authors found no significant difference in the incidence and prevalence of major adverse cardiovascular events, defined as myocardial infarction, transient or permanent cerebrovascular event, or cardiovascular-associated death [43].

However, it is still unknown whether the increased HF risk is similar between RA and PsA. Further studies are warranted to investigate if RA patients really have higher NT-proBNP levels and consequently increased occurrence of subclinical HF than patients with PsA.

Several limitations need to be acknowledged. First, our study has limitations mainly related to the observational design of the study. Weaknesses include lack of a healthy control group. The choice of therapy was made by the rheumatologists and patients, which could result in selection bias or confounding by indication. In our study, patients starting TNFi had significant longer disease duration and had failed synthetical DMARDs before switching. However, withholding treatment with proven efficacy from clinically eligible patients or giving medication with considerable side effects (such as MTX and TNFi) to healthy controls poses ethical problems. Second, the relatively small sample sizes increase the risk of Type-II errors, and might lead to underestimation of real differences and associations. Third, although NT-proBNP is a well-established predictor of future CV risk in general populations with and without CVD, with high sensitivity and specificity and strong positive predictive values [13,14,44], none of the studied patients had clinical HF and the lack of specific test of cardiac function (e.g., echocardiography) can be considered as a limitation.

On the other hand, the strength in the study is a well-characterized study population and design which made it feasible to compare the effect of the two antirheumatic treatment regimens on circulating levels of NT-proBNP in AA.

In conclusion, our data demonstrated an association between inflammation and cardiac function in terms of NT-proBNP. Nevertheless, the results did not show improvement in NT-proBNP levels in response to antirheumatic therapy over 6 months, although there was a small improvement from 6 weeks to 6 months. Importantly, there was no worsening of this surrogate measure of HF, even though inflammatory markers significantly improved. As patients with AA may be at risk for cardiac dysfunction and future CV events, further research is needed to define if NT-proBNP might be a good biomarker for predicting HF in AA patients.

## Supporting information

S1 Table Reference normal values of NT-proBNP. (DOCX)

S1 File Stata file. Stata file containing all data underlying the findings described in this study.
(DTA)
