# Supplementary material for: Antirheumatic therapy is not associated with changes in circulating N-terminal pro-brain natriuretic peptide levels in patients with autoimmune arthritis
Source: PLoS One. 2021 Jun 25;16(6):e0253793. doi: 10.1371/journal.pone.0253793 (PMC8232407; doi:10.1371/journal.pone.0253793)
Supplement: S1 Table — (DOCX) [file pone.0253793.s001.docx]

**S1 Table 1. Reference normal values of NT-proBNP**

Abbreviation: NT-proBNP: N-terminal pro-brain natriuretic peptide
